# Machine learning-based integration develops a neutrophil-derived signature for improving outcomes in hepatocellular carcinoma

**DOI:** 10.3389/fimmu.2023.1216585

**Published:** 2023-07-28

**Authors:** Qiming Gong, Xiaodan Chen, Fahui Liu, Yuhua Cao

**Affiliations:** ^1^ Department of Medical Oncology 2, The People’s Hospital of Guangxi Zhuang Autonomous & Institute of Oncology, Guangxi Academy of Medical Sciences, Nanning, China; ^2^ Department of Nephrology, Affiliated Hospital of Youjiang Medical University for Nationalities, Baise, China; ^3^ Department of Medical Oncology 1, The People’s Hospital of Guangxi Zhuang Autonomous & Institute of Oncology, Guangxi Academy of Medical Sciences, Nanning, China; ^4^ Xiamen Cell Therapy Research Center, The First Affiliated Hospital of Xiamen University, School of Medicine, Xiamen University, Xiamen, China

**Keywords:** neutrophils, HCC, *RTN3*, prognosis, machine learning

## Abstract

**Introduction:**

The heterogeneity of tumor immune microenvironments is a major factor in poor prognosis among hepatocellular carcinoma (HCC) patients. Neutrophils have been identified as playing a critical role in the immune microenvironment of HCC based on recent single-cell studies. However, there is still a need to stratify HCC patients based on neutrophil heterogeneity. Therefore, developing an approach that efficiently describes "neutrophil characteristics" in HCC patients is crucial to guide clinical decision-making.

**Methods:**

We stratified two cohorts of HCC patients into molecular subtypes associated with neutrophils using bulk-sequencing and single-cell sequencing data. Additionally, we constructed a new risk model by integrating machine learning analysis from 101 prediction models. We compared the biological and molecular features among patient subgroups to assess the model's effectiveness. Furthermore, an essential gene identified in this study was validated through molecular biology experiments.

**Results:**

We stratified patients with HCC into subtypes that exhibited significant differences in prognosis, clinical pathological characteristics, inflammation-related pathways, levels of immune infiltration, and expression levels of immune genes. Furthermore, A risk model called the "neutrophil-derived signature" (NDS) was constructed using machine learning, consisting of 10 essential genes. The NDS's RiskScore demonstrated superior accuracy to clinical variables and correlated with higher malignancy degrees. RiskScore was an independent prognostic factor for overall survival and showed predictive value for HCC patient prognosis. Additionally, we observed associations between RiskScore and the efficacy of immune therapy and chemotherapy drugs.

**Discussion:**

Our study highlights the critical role of neutrophils in the tumor microenvironment of HCC. The developed NDS is a powerful tool for assessing the risk and clinical treatment of HCC. Furthermore, we identified and analyzed the feasibility of the critical gene *RTN3* in NDS as a molecular marker for HCC.

## Background

Hepatocellular carcinoma (HCC), also known as liver cancer, is a common malignancy with a high incidence rate. Drugs such as sorafenib and lenvatinib (1), are widely used in the treatment of HCC, and new drugs like atezolizumab combined with bevacizumab and sintilimab combined with bevacizumab are being developed (2, 3). These drugs target specific populations, with some suitable for patients with unresectable HCC who have not undergone systemic treatment, like doxorubicin and lenvatinib (4, 5), while others are appropriate for patients with HCC who have received specific treatments, like regorafenib and cabozantinib (6). Despite promising results in clinical trials, these treatment methods only benefit a small proportion of patients, highlighting critical clinical challenges. Therefore, selecting the most appropriate treatment plan based on the specific conditions of the patients and the target population of the drug is crucial in the treatment of HCC. Advancements in biotechnology, particularly high-throughput sequencing technologies, have deepened our understanding of tumor molecular subtyping, enabling tumor treatment based on molecular subtypes. Gene-based molecular subtyping has emerged as a new approach to the treatment of tumors. Scientists have successfully developed personalized treatment plans based on molecular subtyping for various cancer types. For example, the *PAM50* gene subtyping technology has been successfully applied in chemotherapy decision-making for the treatment of breast cancer (7). *EGFR* gene mutation subtyping has also been widely adopted in the treatment of lung cancer for selecting targeted drugs against *EGFR (* 8). Similarly, *BRAF* gene mutation subtyping has found extensive application in personalized treatment plans for colon cancer and melanoma (9, 10). These accomplishments indicate that gene-based molecular subtyping technologies will be crucial in future treatments of tumors, offering patients more accurate and effective treatment options.

The latest research has unveiled the immune microenvironment subtypes of HCC through large-scale single-cell sequencing and provided an in-depth analysis of the functional heterogeneity of tumor-associated neutrophils. This study demonstrates that targeting tumor-associated neutrophils may emerge as a new immunotherapy strategy for HCC (11). Neutrophils play a crucial role in the immune system by regulating immune responses, combating infections, and maintaining tissue homeostasis. Recent studies have indicated that neutrophil-mediated immune processes, known as neutrophil extracellular traps (NETs), have a significant impact on the development of tumors as they serve as a vital step in innate and adaptive immune responses triggered by infectious and sterile stimuli (12). Previous studies suggested that cancer-induced NETs primarily function in the circulation, promoting cancer-related thrombosis (13). Subsequent studies have revealed that NETs influence every stage of the metastatic cascade, including the progression, invasion, and migration of primary tumors, survival in circulation, chemoattraction to secondary sites, extravasation, colonization, and growth of metastatic tumor cells (14). These findings highlight the fact that the functional transformation of neutrophil subtypes in the tumor microenvironment is influenced by the specific characteristics of the tumor microenvironment, though the precise mechanisms remain unclear (15). In summary, neutrophils play pivotal roles in the development, metastasis, treatment, and immune evasion of HCC.

The advancement of single-cell research technology has brought about the ability to accurately analyze the heterogeneity of the tumor microenvironment in different clinical types of HCC and discern distinct subtypes of neutrophils with unique characteristics during the development of the tumor. These findings have been instrumental in uncovering the dynamic changes in levels of gene expression within these neutrophil subtypes, shedding light on the molecular mechanisms underlying the development of tumors, and identifying potential targets for diagnosis and treatment. However, it is important to note that neutrophils are fragile cell types that can easily be lost during tissue dissociation. Moreover, neutrophils have a limited number of expressed genes and tend to exhibit low expression levels of characteristic genes, further complicating the analysis of their cell subtypes and gene expression profiles. Additionally, the high cost associated with single-cell sequencing technology poses a significant barrier to its widespread clinical application for studying neutrophils. Nonetheless, it is feasible to differentiate patients with HCC based on neutrophils, thereby identifying subtypes and evaluating patient prognosis for clinical treatment and medication guidance. It is crucial to find a simple and effective method to describe the “neutrophil characteristics” of patients with HCC. With the advancements in bioinformatics technology, several prognostic gene characteristics have been developed (16, 17). In the case of HCC, numerous multi-gene signature characteristics, such as the well-known ferroptosis signature (18), m6A signature (19), and others (20), have been discovered to assess patient risk. However, the efficacy of these multi-gene expression signatures can be challenging to validate and apply effectively due to single-machine learning and inappropriate statistical methods.

In this study, we used machine learning to develop and validate risk stratification characteristics for patients with HCC using neutrophil-related characteristic markers. We assessed the value of different risk stratifications in terms of biological and clinical pathological characteristics, prognosis, and their application in immunotherapy and targeted chemotherapy treatments across four independent public datasets. Furthermore, based on the analysis results, this study verified a new molecular marker for HCC. Overall, this study aims to optimize precision treatment and enhance the clinical outcomes of patients with HCC.

## Materials and methods

### Data resources

High-throughput sequencing data in TPM format for HCC were obtained from The Cancer Genome Atlas (TCGA) database, along with corresponding clinical phenotype data. We excluded samples that lacked survival time or status and retained only those with a survival time greater than 0 days. This resulted in 365 tumor samples. Similarly, we obtained another HCC high-throughput sequencing dataset, HCCDB18, from http://lifeome.net/database/hccdb/download.html. We removed normal samples to retain only tumor tissue and obtained survival data for all patients with a survival time greater than 0 days. This yielded a final set of 212 tumor tissues. For the datasets GSE14520 and GSE116174, we obtained expression profile data and survival times from the Gene Expression Omnibus (GEO) database of the National Center for Biotechnological Information (NCBI) database. We excluded samples lacking survival time or status and included all patients with a survival time greater than 0 days in the analysis. We downloaded platform files and converted probes to gene names. We removed data with one probe corresponding to multiple gene names and averaged data with multiple probes corresponding to a single gene. Ultimately, we identified 242 tumor tissues from the GSE14520 dataset and 64 tumor tissues from the GSE116174 dataset. Additionally, we obtained single-cell sequencing data for HCC (Accession number: GSE215428) from the GEO database.

### Dimensionality reduction and cell annotation of single-cell clusters

First, we filtered the single-cell data that required each gene to be expressed in a minimum of three cells, while each cell had to express at least 250 genes. Additionally, we used the PercentageCharacteristicset function to calculate the proportions of mitochondrial and rRNA genes, ensuring that each cell expressed fewer than 2000 genes. Subsequently, we performed log-normalization on the data from six samples to standardize them. To identify highly variable genes, we utilized the FindVariableCharacteristics function, employing variance stabilization transformation (“vst”). All genes were then scaled using the ScaleData function, followed by dimensionality reduction using RunPCA to identify anchor points. The clustering of cells was achieved through the utilization of the FindNeighbors and FindClusters functions, and classical marker genes were used for cell annotation. The clusterProfiler package was used for the Kyoto Encyclopedia of Genes and Genomes (KEGG) pathway enrichment analysis of the marker genes across different subgroups.

### Construction of molecular subtypes and risk model

Using single-cell analysis, 208 marker genes specific to neutrophils were identified. The ConsensusClusterPlus package in R was used to cluster patients based on the expression of these neutrophil marker genes in tumor tissues from the TCGA dataset. The partition around medoids (PAM) algorithm was used, with “pearson” serving as the distance metric. We performed 500 bootstraps, each including 80% of the patients from the training set. Clustering numbers ranging from 2 to 10 were set, and the optimal classification was determined by evaluating the consensus matrix and cumulative distribution function.

Based on the neutrophil marker genes, univariate Cox analysis was conducted to select prognostic-related genes with a P-value of <0.001. These genes were further integrated into a high-precision and stable model using our machine learning-based integration program. For the TCGA dataset, we fitted 101 prediction models using the LOOCV framework and calculated the concordance index (C-index) of each model on all validation datasets. The model with the highest average C-index was considered the best.

### Analysis and comparison of biological features

We compared different cell scores among the three subtypes using the ESTIMATE algorithm, the MCPcounter package, and the CIBERSORT algorithm. To calculate the scores of 28 immune cells, we used single-sample gene set enrichment analysis (ssGSEA) with 28 characteristic genes of immune cells obtained from previous research (21). Additionally, the tumor immune dysfunction and exclusion (TIDE) software was used to evaluate the potential clinical effects of immunotherapy and risk models. To assess the scores of relevant pathways, we obtained the inflammatory pathway gene set from the KEGG website and calculated pathway scores using the ssGSEA method. Furthermore, the patient scores for KEGG database-related pathways were determined using the gene set variation analysis (GSVA) package in R, with gene sets downloaded from the GSEA website. The maftools package showed the top 20 mutated genes and generated a waterfall chart. The copy number variation (CNV) dataset was also obtained and analyzed to determine the proportion of deleted or amplified genes. To explore potential therapeutic targets for high- and low-risk groups, we used the Cancer Cell Line Encyclopedia (CCLE) database of drug-sensitive cell lines as the training set. Using the Cancer Therapeutics Response Portal (CTRP) and Profiling Relative Inhibition Simultaneously in Mixture (PRISM) methods, we predicted the drug sensitivity of each patient in the TCGA dataset. Potential regulatory drugs were screened based on |log2(Fold Change [FC])| >0.2.

### Cell culture and transfection

The human HCC cell line HepG2 (KCB200507YJ) was obtained from the Chinese Academy of Sciences. The cells were cultured in Dulbecco’s Modified Eagle’s Medium (Gibco, Carlsbad, CA, USA) supplemented with 8.0% fetal bovine serum. To silence the expression of *RTN3*, HepG2 cells were transfected with small interfering RNA (siRNA) using hU6-MCS-CBh-gcGFP-IRES-puromycin (Shanghai Gene Chem Co., Ltd.). The HepG2 cells were divided into two groups: the control group and the si-RTN3 group.

### Western blot assay

To obtain total cellular proteins from HepG2 cells, radioimmunoprecipitation assay buffer (RIPA) lysate (Beyotime, Shanghai, China) was used, and protein quantification was performed using the bicinchoninic acid (BCA) assay kit (Servicebio, Wuhan, China). Cell samples containing 30 μg of total protein were loaded onto sodium dodecyl sulfate-polyacrylamide gel electrophoresis (SDS-PAGE) and subsequently transferred to polyvinylidene fluoride (PVDF) membranes. The membranes were then incubated overnight at 4°C with anti-RTN3 (Abcam, Cat# Ab68328) and anti-β-tubulin (Affinity Biosciences, Cat# T0023). Subsequently, the membranes were incubated with goat anti-rabbit immunoglobulin G (IgG; S0001, 1:5000, Affinity Biosciences) and goat anti-mouse IgG (S0002, 1:5000, Affinity Biosciences) for 50 minutes and visualized using Tanon-5200 (Tanon, Shanghai, China). Further details regarding these experimental procedures have been described previously (22).

### Colony formation and Transwell assay

For colony formation, cells were directly seeded into 6-well plates at a density of 3 × 10^2^ cells per well. After 14 days, the wells were rinsed three times with phosphate-buffered saline (PBS) at room temperature. Subsequently, cells were stained with paraformaldehyde (1 ml/well) and incubated with crystal violet solution (1 ml/well) for 30 minutes. In the Transwell assay, 8-μm Transwell chambers (Corning, USA) were used. The upper chamber, pre-coated with Matrigel (Corning, USA), was used for cell plating, while the lower chamber was filled with a complete medium. After fixing the cells with paraformaldehyde, they were stained with a 0.1% crystal violet solution for five minutes and left to dry overnight. The specific steps of the Transwell assay were conducted as described previously (23).

### Statistical analysis

Statistical analysis was conducted using R software (version 4.0.5). Spearman’s correlation coefficient was used to evaluate the correlation between two continuous variables. The chi-square test was used to compare categorical variables, while the Wilcoxon rank sum test, or t-test, was used for comparing continuous variables. A significance level of P <0.05 was used to determine statistical significance for all tests.

## Results

### Dimensionality reduction and clustering of single cells

After applying quality control measures and filtering, a total of 17,277 cells were obtained. The statistical analysis of cell numbers before and after filtering is shown in **Figure S1A**. To reduce dimensionality and identify anchor points, we performed Principal Component Analysis (PCA) using the RunPCA method (**Figure S1B**). Additionally, t-distributed Stochastic Neighbor Embedding (t-SNE) analysis was conducted on the 17,277 cells using the Runt-SNE function, and **Figure S1C** shows the t-SNE cell distribution maps for the six samples. For clustering analysis, we used the FindNeighbors and FindClusters functions with a resolution set at 0.2 and a dimensionality of 20. As a result, we identified 10 distinct subpopulations. Cell annotation was carried out using established marker genes, wherein subpopulations 0, 1, 2, and 4 exhibited expression of T-cell markers CD2, CD3D, CD3E, and CD3G, respectively. Subpopulation 6 showed expression of the B-cell markers CD19, CD79A, and MS4A1. The dendritic cell marker CLEC4C was expressed in subpopulation 9, while neutrophil markers CSF3R, S100A8, and S100A9 were found in subpopulations 3, 7, and 8, respectively (**Figure S1D**).

**Figure 1:**
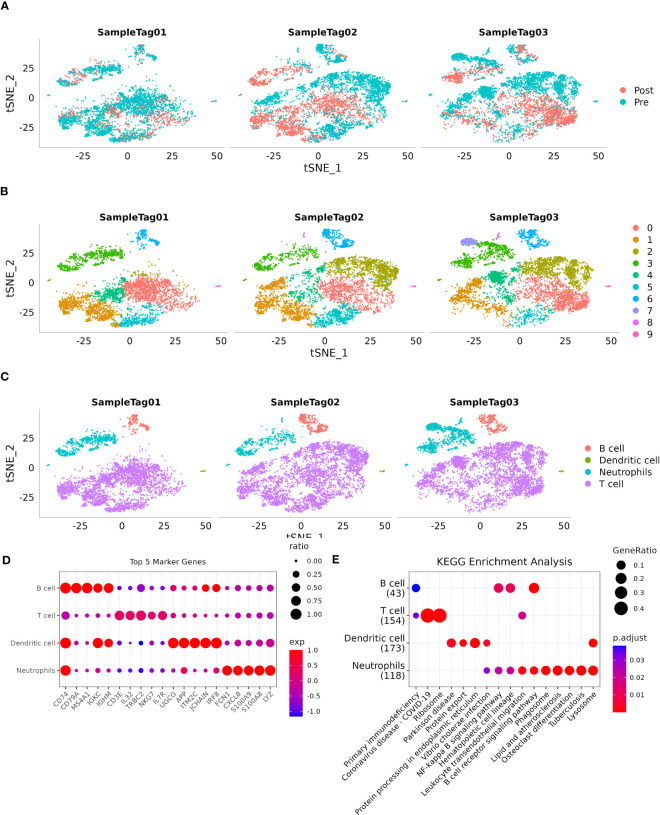
Single-cell landscape of patients with HCC. **(A)**: Distribution of each sample shown on a t-SNE plot; **(B)**: Distribution of 10 subtypes shown on a t-SNE plot; **(C)**: Subtypes after cell annotation shown on a t-SNE plot; **(D)**: Expression of the top five marker genes of annotated subtypes illustrated on a dot plot; **(E)**: KEGG enrichment analysis of annotated subtypes visualized on a dot plot.

**Figure 1A** shows a t-SNE distribution map depicting different sample populations. **Figure 1B** shows a t-SNE distribution map specifically focusing on the 10 subpopulations. Furthermore, **Figure 1C** shows an annotated t-SNE distribution map highlighting the subpopulations. To identify marker genes within these subpopulations, the FindAllMarkers function was employed with specific parameters, including a logFC of 0.5 and a minimum percentage of differentially expressed genes (Minpct) of 0.35. This analysis yielded four subpopulations with a corrected P-value of <0.05. **Figure 1D** shows the expression of the top five significant marker genes in each of these subpopulations. Detailed information about the marker genes is provided in scRNA_marker_gene.txt (Table). Furthermore, KEGG annotation was conducted on the marker genes from the four subpopulations. The results revealed their involvement in various functions and disease pathologies, highlighting the vital role of immune cells in maintaining overall health (**Figure 1E**).

### Construction of molecular subtypes

**Figure 2:**
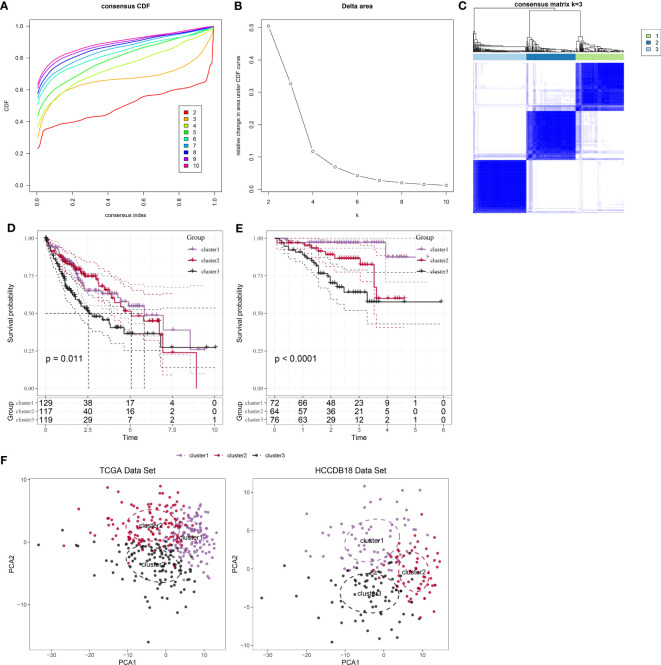
Identification and analysis of subtypes with neutrophil characteristics in patients with HCC. **(A)**: CDF curve of samples from the TCGA dataset. **(B)**: Delta area curve of consensus clustering for samples from the TCGA dataset, showing the relative change in the area under the CDF curve for each category number “k” compared to “k-1.” The horizontal axis represents the category number “k,” while the vertical axis represents the relative change in the area under the CDF curve. **(C)**: Heatmap showing the sample clustering at consensus “k = 3.” **(D)**: KM curves demonstrating the prognosis of three subtypes in the TCGA dataset. **(E)**: KM curves demonstrating the prognosis of three subtypes in the HCCDB18 dataset. **(F)**: PCA showing the distribution of three subtypes in the TCGA and HCCDB18 datasets.

Following the utilization of 208 markers specific to neutrophils, we proceeded to construct molecular subtypes. To determine the optimal number of clusters, we used cumulative distribution function (CDF) analysis. The CDF Delta area curve indicated that a cluster selection of 3 yielded relatively stable clustering results (**Figures 2A, B**). Consequently, we chose a “k” value of 3 to define three distinct molecular subtypes (**Figure 2C**). Notably, these three subtypes showed significant differences in prognosis (**Figure 2D**, P = 0.011), with patients in cluster 3 exhibiting the poorest prognosis. Similarly, when applying the same methodology to the HCCDB18 dataset, we obtained three subtypes with comparable prognostic implications (**Figure 2E**; P <0.0001). Detailed information about the molecular subtypes for both datasets can be found in tables tcga.subtype.cli.txt and HCCDB18.subtype.cli.txt. Furthermore, we conducted PCA analysis based on the marker genes specific to neutrophils, generating a scatter plot that illustrates the distribution of the three subtypes as shown in **Figure 2F**. Our analysis suggests that the significant heterogeneity observed among patients with HCC may be attributed to distinct “neutrophil characteristics.”

### Clinical features of molecular subtypes

**Figure 3:**
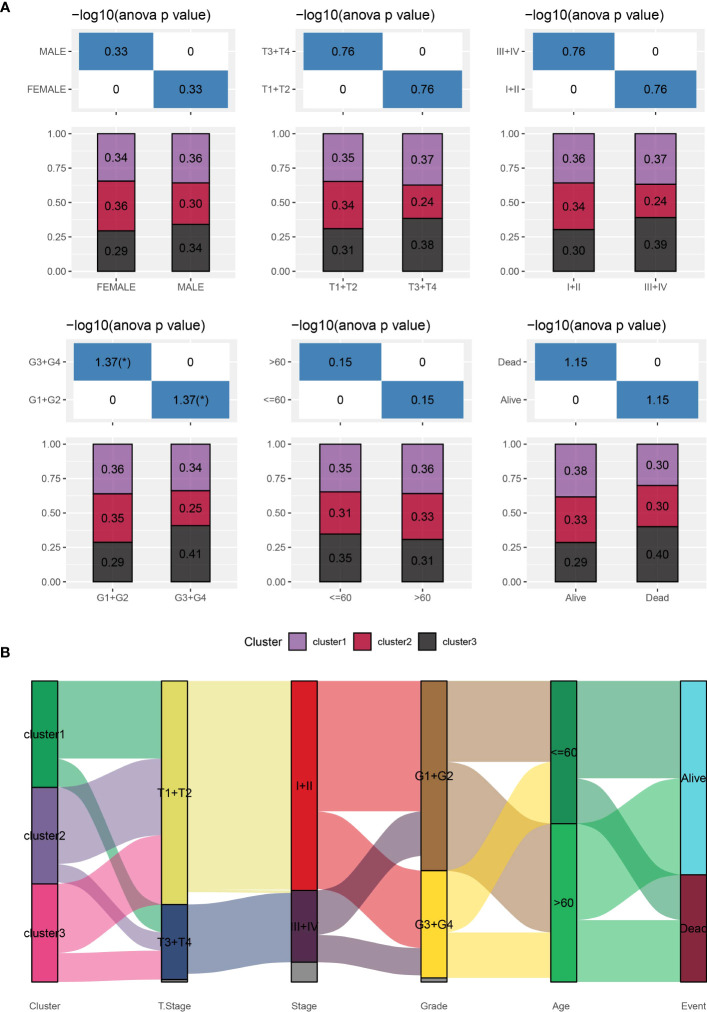
Distribution of clinical characteristics across different subtypes. **(A)**: Sample distribution of clinical characteristics across different subtypes in the TCGA-LIHC cohort. The horizontal axis represents the different sample groups, while the vertical axis represents the percentage of clinical information within the corresponding group samples. Different colors represent different molecular subtypes. **(B)**: Sankey Diagram showing the association between different subtypes and clinicopathological characteristics in patients with HCC.

Furthermore, we conducted a comprehensive analysis of the clinical and pathological characteristics of different molecular subtypes in the TCGA dataset. Specifically, we compared the distribution of various clinical characteristics among the three molecular subtypes to identify potential differences. In our analysis, while applying a chi-square test, we found that cluster 3 samples exhibited a higher proportion of patients with G3 plus G4 stages compared to the other subtypes. This finding suggests a potential association between molecular subtypes and tumor grade (**Figure 3**).

### Functional analysis of immune-related pathways among molecular subtypes

**Figure 4:**
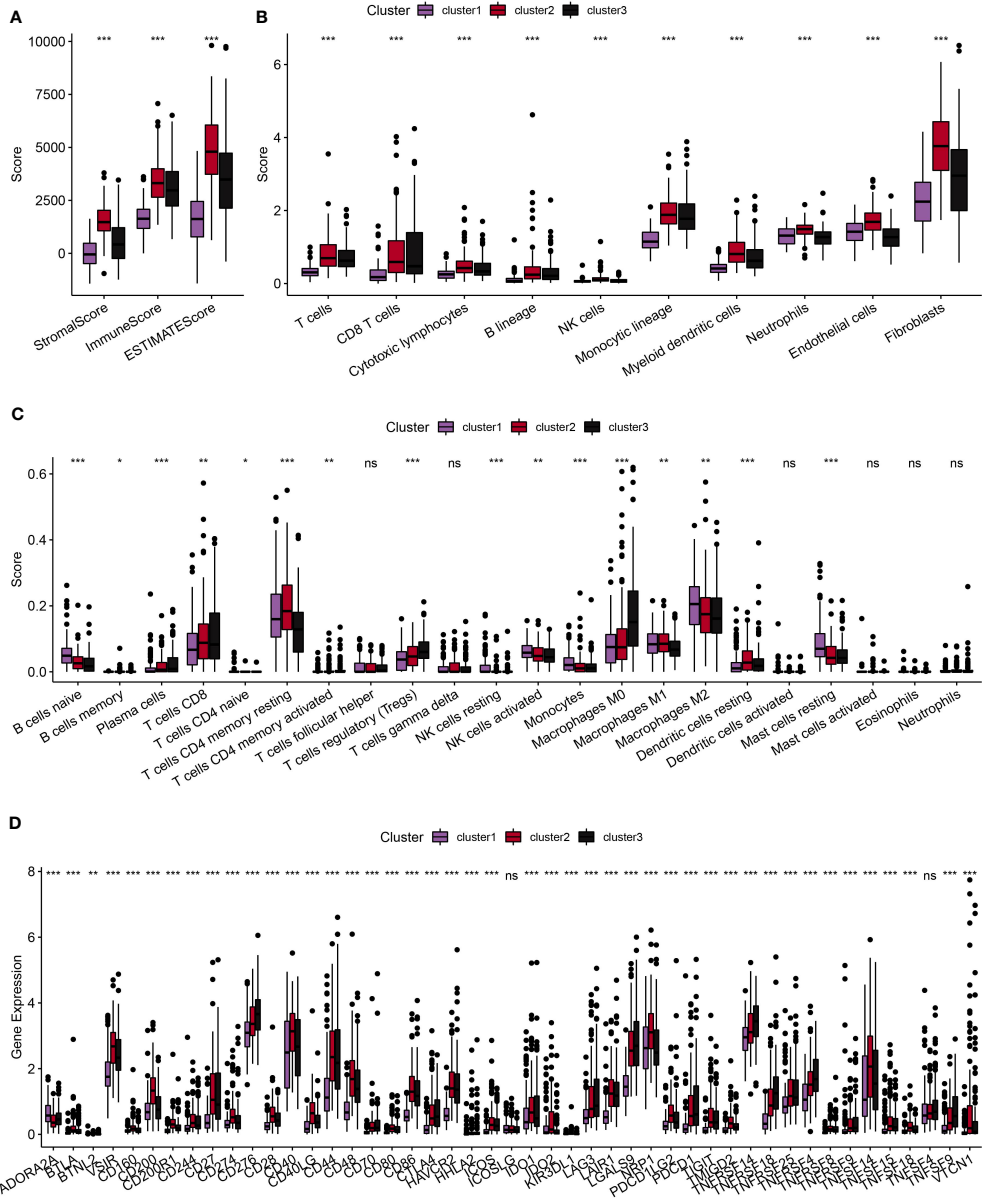
Comparative analysis of immune characteristics among different subtypes. **(A)**: Comparative analysis of immune characteristics among different subtypes in the TCGA dataset, focusing on the predicted immune scores by ESTIMATE. **(B)**: Comparative analysis among different subtypes in the TCGA dataset, examining the scores of 10 predicted immune cell types using the MCPcounter method. **(C)**: Comparative analysis of immune characteristics among different subtypes in the TCGA dataset of scores of 22 predicted immune cell types using the CIBERSORT algorithm. **(D)**: Comparative analysis of immune characteristics among different subtypes in the TCGA dataset, highlighting the expression of immune checkpoints across the three subtypes. ns, p ≥ 0.05; *, p < 0.05; **, p < 0.01; ***, p < 0.001; ****, p < 0.0001.

First, we used the ESTIMATE algorithm to calculate the immune scores of patients. The comparison showed that clusters 2 and 3, which were associated with a poor prognosis, exhibited higher immune cell scores (**Figure 4A**). Subsequently, we used the MCPcounter package to calculate scores for 10 different types of immune cells. These results also indicated that clusters 2 and 3 showed higher immune cell scores (**Figure 4B**). Furthermore, we used the Cell-type Identification by Estimating Relative Subsets of RNA Transcripts (CIBERSORT) method to calculate scores for 22 different types of immune cells. This analysis demonstrated significant differences in the majority of immune cell types among the three subtypes (**Figure 4C**). Moreover, we conducted a comparison of the expression levels of immune checkpoint genes. With the exception of *TNFSF4* and *ICOSLG*, the majority of the immune checkpoint genes showed varying expression levels among the three subtypes. Notably, clusters 2 and 3 showed higher levels of immune checkpoint gene expression (**Figure 4D**). In summary, our comprehensive analyses indicated that clusters 2 and 3, which were associated with a poor prognosis, showed higher levels of immune infiltration.

### Analysis of inflammatory pathways among molecular subtypes

**Figure 5:**
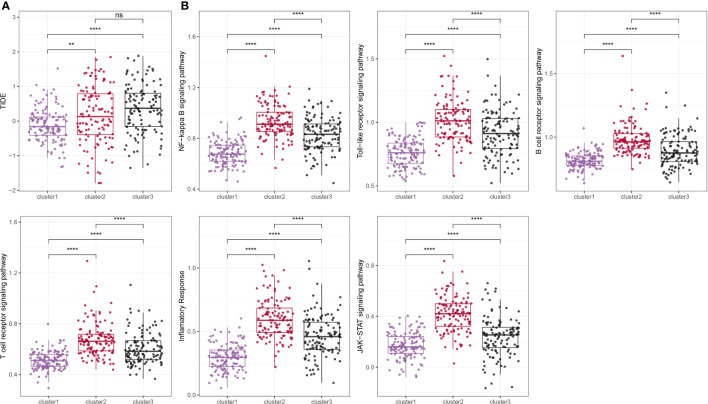
Comparison of TIDE score and inflammation-related pathway score among different subtypes. ns, p ≥ 0.05; **, p < 0.01; ****, p < 0.0001.

We employed the TIDE online tool to predict the likelihood of immune evasion in patients, where a higher TIDE score indicates a more significant potential for immune evasion. As shown in **Figure 5A**, clusters 2 and 3, which were associated with poor prognoses, showed higher TIDE scores compared to cluster 1, suggesting a greater tendency for immune evasion. Since the molecular subtypes constructed were closely associated with the immune system, we acquired inflammation-related pathway gene sets from the KEGG website and calculated the pathway scores using the ssGSEA method. As shown in **Figure 5B**, we observed that cluster 1 had significantly lower inflammatory pathway scores compared to the other subtypes.

### KEGG pathway analysis of molecular subtypes

**Figure 6:**
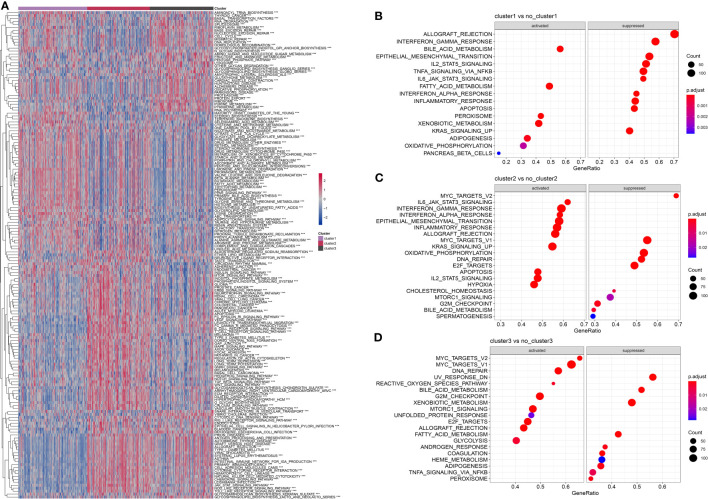
Comparison of pathway characteristics among different subtypes in the TCGA dataset. **(A)**: A heatmap showing the enrichment scores for enriched pathways in three subtypes of the TCGA dataset. **(B)**: A bubble plot showing the enriched pathways in cluster 1 of the TCGA dataset. **(C)**: A bubble plot showing the enriched pathways in cluster 2 of the TCGA dataset. **(D)**: A bubble plot showing the enriched pathways in cluster 3 of the TCGA dataset. ***, p < 0.001.

To explore the heterogeneity of patients with HCC, we obtained KEGG pathway-related gene sets from the GSEA website and calculated pathway scores for each patient using the R package GSVA. By analyzing these scores, we identified multiple pathways that showed significant differences among the three subtypes of HCC, as shown in **Figure 6A**. Further details and the results of our analysis are summarized in pathwy_p_fit.txt. Additionally, we conducted a comparison of differential gene expression among the different subtypes and performed GSEA analysis using the R package clusterProfiler. **Figures 6B–D** show the patterns of pathway activation and suppression observed across the distinct subtypes of HCC. In summary, our findings demonstrated that marker genes associated with neutrophils effectively distinguished the heterogeneity of patients with HCC. Intriguingly, these marker genes suggested the presence of “neutrophil characteristics” among patients with different subtypes of HCC.

### Construction of a neutrophil-derived signature and investigation of the role of *RTN3* in HCC

**Figure 7:**
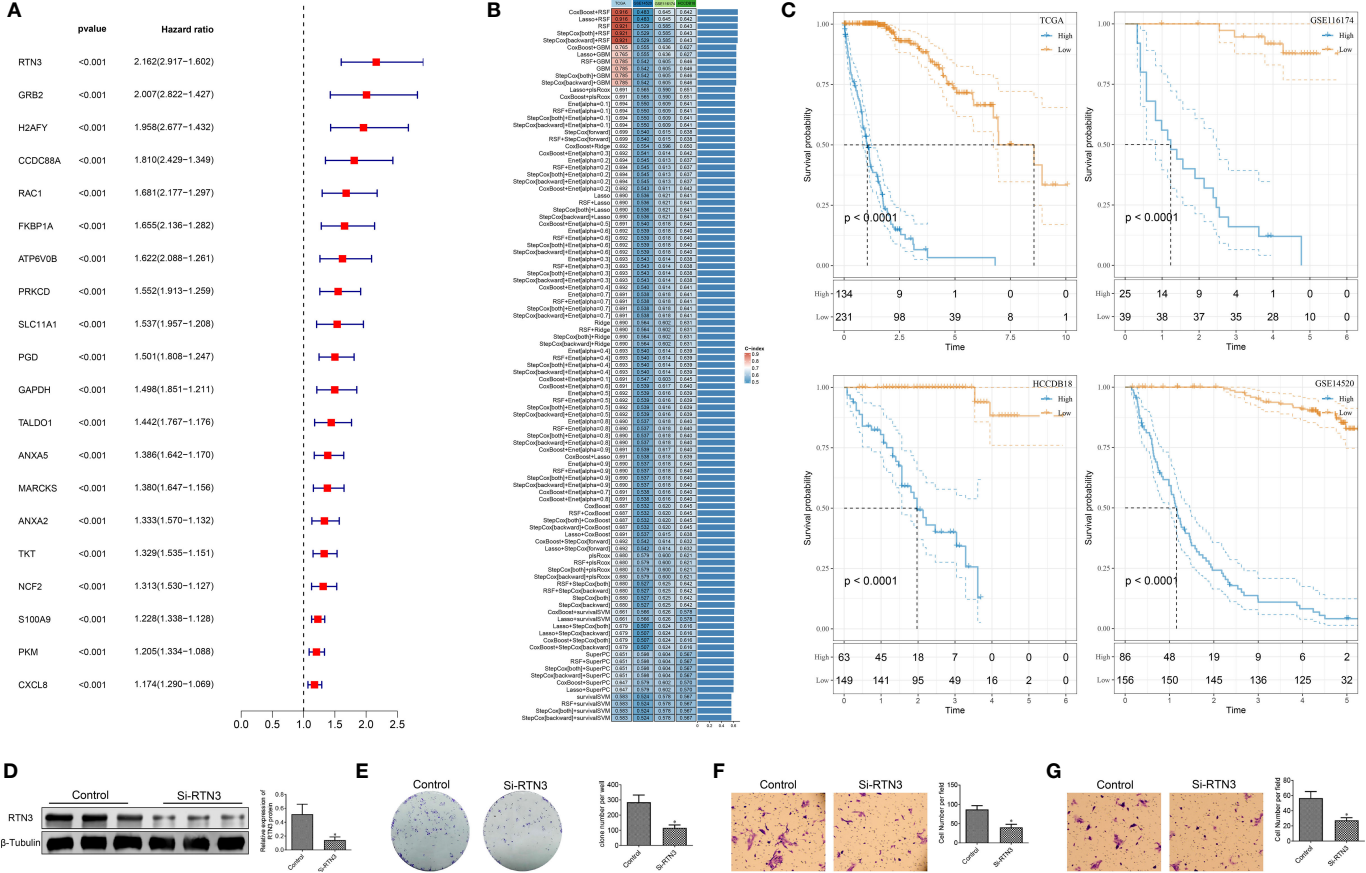
Construction of the prognostic model based on machine learning and biological functional analysis of RTN3. **(A)**: A forest plot showing prognostic-related genes identified in the analysis. **(B)**: Optimal combination of machine learning-based feature selection for constructing the risk model. **(C)**: Kaplan-Meier curves demonstrating the high- and low-risk groups in the training and validation sets. **(D)**: Western blot analysis showing the expression of RTN3 protein in HepG2 cells after transfection of si-DUSP1. **(E)**: Assessment of the proliferation activity of HepG2 cells using a colony formation assay. **(F, G)**: Evaluation of migration and invasion abilities of HepG2 cells using a Transwell assay. *, p < 0.05.

Based on the identified “neutrophil characteristics” among patients with HCC, we conducted an analysis to identify prognosis-related genes. Using univariate Cox regression analysis with a significance level of P <0.001, we identified 20 genes, as shown in **Figure 7A**. These genes were derived from marker genes based on neutrophils and obtained from the TCGA database. To develop a consistent prognostic model, we used a machine learning-based integration program, using the 20 identified genes as input characteristics. Specifically, we fitted 101 prediction models using the Leave One Out Cross-Validation (LOOCV) framework. We calculated the C-index of each model across all validation datasets, as shown in **Figure 7B**. The optimal model, which combined CoxBoost and RSF, yielded the highest average C-index of 0.671.

Further analysis focused on 10 critical genes, such as *ANXA5, ATP6V0B, GAPDH, GRB2, PRKCD, RAC1, RTN3, S100A9, TALDO1*, and *TKT*. We examined the expression levels of these genes in both the TCGA dataset and other validation sets. By employing the rfsrc function, we predicted the risk score for each patient based on the expression levels of these 10 genes. Subsequently, we standardized the risk scores into z-scores. Using a cutoff of 0, we divided patients into high- and low-risk groups within different datasets, including GSE14520, GSE116174, HCCDB18, and TCGA-LIHC, as shown in **Figure 7C**. In summary, our findings suggest that this 10-gene signature could serve as a robust prognostic tool for patients with HCC.

The significant expression differences of *RTN3* in multiple HCC cohorts indicate an association between its expression level and HCC patient prognosis(**Figure S2**). We conducted an experiment using siRNA to manipulate the levels of *RTN3* in HepG2 cells. In comparison to the control group, the si-RTN3 group showed a significant decrease in the expression of the *RNT3* protein, as shown in **Figure 7D**. The colony formation assay showed that the proliferation ability of HepG2 cells was significantly inhibited in the si-RTN3 group compared with the control group (**Figure 7E**). Additionally, the Transwell assay demonstrated that there was a decrease in the number of migrated and invaded cells in the si-RTN3 group compared to the control group (**Figures 7F, G**). Overall, our findings indicate that the knockdown of *RTN3* suppressed the proliferation, migration, and invasion of HepG2 cells.

### Comparison of RiskScore based on different clinical characteristics

**Figure 8:**
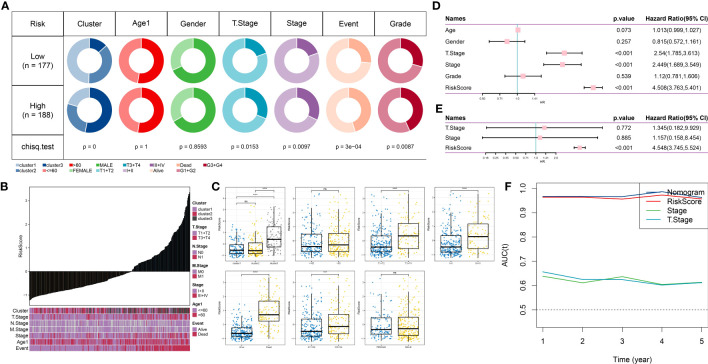
Clinical applications of the prognostic model. **(A)**: Comparison of different clinical characteristics between high-risk and low-risk groups. **(B)**: Distribution of different clinical characteristics with increasing risk scores. **(C)**: Comparison of risk scores among different clinical characteristics. **(D)**: Forest plot of univariate Cox analysis for clinical characteristics. **(E)**: Forest plot of multivariate Cox analysis for clinical characteristics. **(F)**: Trend of changes in AUC for T-stage, Stage, and Risk Score for one to five years. ns, p≥ 0.05; ***, p < 0.001; ****, p < 0.0001.

To examine the association between RiskScore and the clinical characteristics of tumors, we conducted an analysis using the TCGA dataset. Our findings revealed a positive correlation between clinical grade and risk score (**Figures 8A, B**). Additionally, we compared the high and low-risk scores across different clinical grades and observed that patients with higher clinical grades showed higher risk scores (**Figure 8C**). Subsequently, we performed both univariate and multivariate Cox regression analyses to investigate the prognostic significance of these clinical characteristics, as shown in **Figures 8D, E**. The results indicated that T-stage (P <0.001), Stage (P <0.001), and RiskScore (P <0.001) were all associated with prognosis and served as independent risk factors. However, the multivariable Cox regression analysis revealed that only RiskScore (P <0.001) remained significantly associated with prognosis. Additionally, we constructed a nomogram incorporating RiskScore, T-stage, and Stage. We assessed its performance by calculating the area under the curve (AUC) value and found that its predictive accuracy was similar to that of RiskScore alone (**Figure 8F**). These findings indicate that our RiskScore-based model holds significant prognostic value for patients.

### Mutation features of the prognostic model

**Figure 9:**
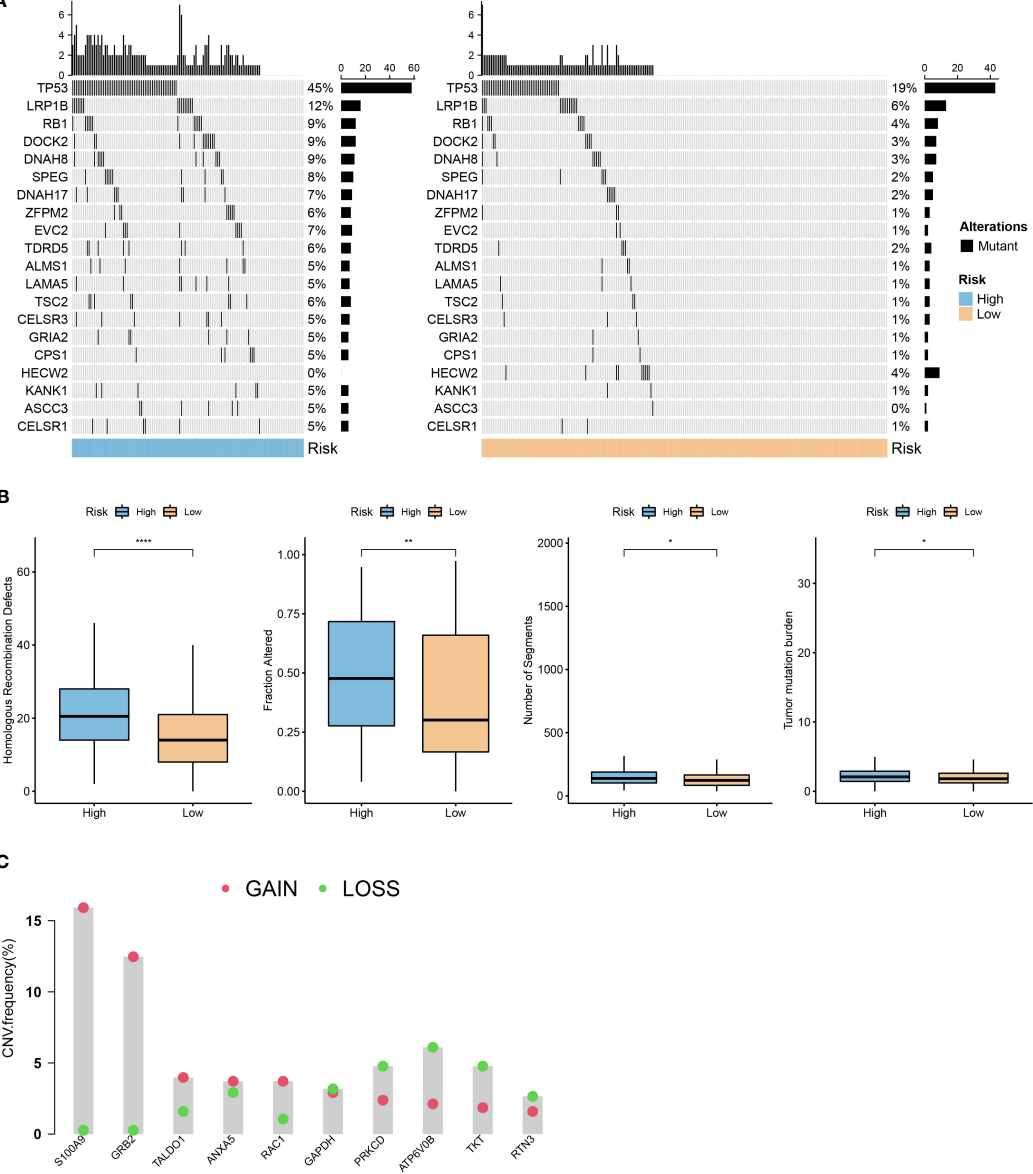
Mutation characteristics of the prognostic model. **(A)**: A waterfall plot of the top 20 gene mutations in high-risk and low-risk groups. **(B)**: Comparison of differences in homologous recombination defects, fraction altered, number of segments, and tumor mutation burden between high- and low-risk groups in the TCGA dataset. **(C)**: Distribution of the proportion of patients with gene CNV mutations in the TCGA dataset module. *, p < 0.05; **, p < 0.01; ****, p < 0.0001.

Using the R language maftools package, we generated a waterfall plot showing the top 20 genes with mutations. The data showed higher mutation frequencies in the high-risk group compared to the low-risk group (**Figure 9A**). Furthermore, we conducted a comparison between the high-risk and low-risk groups, examining the distribution of homologous recombination defects (P <0.001), fraction altered (P <0.001), number of segments (P <0.001), and tumor mutation burden (P <0.001). As shown in **Figure 9B**, there were significant differences in fraction altered, number of segments, and tumor mutation burden between the high- and low-risk groups. We also obtained CNV data and showed the proportions of deletions and amplifications for the 10 genes used in constructing the risk model (**Figure 9C**).

### Immune features of the prognostic model

**Figure 10:**
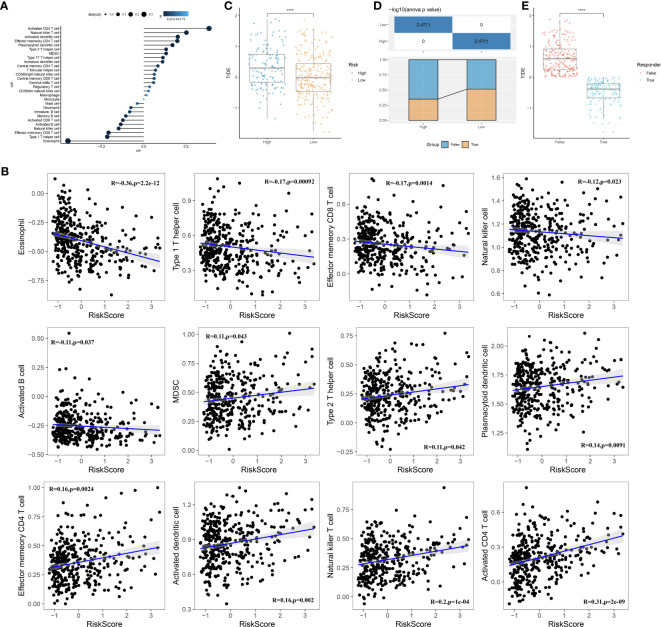
Immune characteristics of the prognostic model. **(A)**: Correlation analysis between the scores of 28 immune cells and risk scores. **(B)**: A scatter plot analysis of the correlation between risk scores and immune cells. **(C)**: Comparison of risk scores with TIDE scores. **(D)**: Comparison of the distribution of immune therapy response, non-response, and high- or low-risk groups. **(E)**: Comparison of TIDE scores between the immune therapy response and non-response groups. ****, p < 0.0001.

We conducted an analysis to examine the correlation between RiskScore and 28 immune cells using the ssGSEA method (**Figure 10A**). Notably, several immune cells showed a significant correlation with the RiskScore. To provide a visual representation of these correlations, scatter plots were generated to depict the correlation between 12 immune cells and RiskScore (**Figure 10B**). Furthermore, we used the TIDE software (available at http://tide.dfci.harvard.edu/) to assess the potential clinical effects of immune therapy in conjunction with our risk model. Higher TIDE prediction scores indicate a greater likelihood of immune evasion and a reduced possibility of benefiting from immune therapy. As shown in **Figure 10C**, patients with a high RiskScore tended to have higher TIDE prediction scores, suggesting a diminished likelihood of benefiting from immune therapy. Furthermore, our analysis revealed a higher proportion of high-risk patients in the non-responsive group compared to the responsive group (**Figure 10D**). Notably, the non-responsive group exhibited higher TIDE prediction scores (**Figure 10E**). These findings collectively indicate that our RiskScore-based model has the ability to predict the response to immune therapy and identify patients who may not derive substantial benefits from it.

### Identification of potential therapeutic drugs for HCC

**Figure 11:**
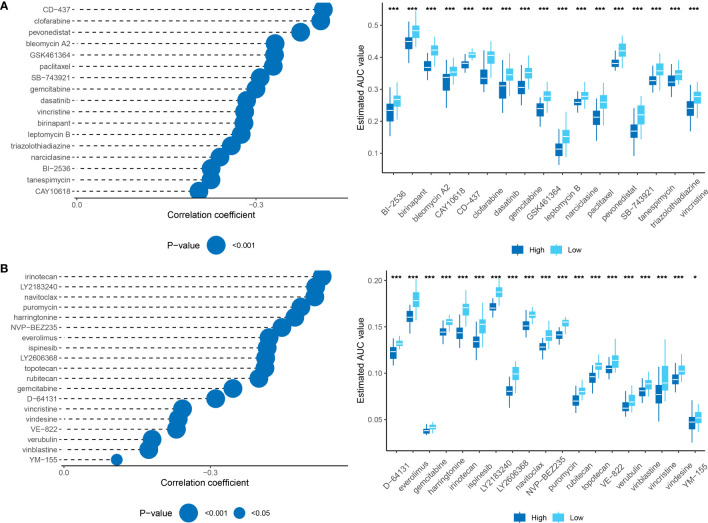
**(A)**: Results of Spearman’s correlation analysis and differential drug response analysis of CTRP-derived compounds. **(B)**: Results of Spearman’s correlation analysis and differential drug response analysis of PRISM-derived compounds. Note that lower values on the y-axis of boxplots indicate greater drug sensitivity. *, p < 0.05; ***, p < 0.001.

To identify candidate drugs with higher drug sensitivity, we employed two distinct approaches using drug response data from the Cancer Therapeutics Response Portal (CTRP) and Profiling Relative Inhibition Simultaneously in Mixture (PRISM) datasets. First, we conducted a differential drug response analysis by comparing the top 10% and bottom 10% groups based on the pharmacological profiling score (PPS). This analysis allowed us to identify compounds with log2FC >0.10 that exhibited lower AUC estimates in the high RiskScore group. Second, we conducted a Spearman correlation analysis between the AUC values and the RiskScore. We selected compounds that showed negative correlation coefficients (Spearman’s r for CTRP and PRISM, <-0.10 and <-0.1, respectively). The results from both approaches consistently demonstrated that all identified compounds had lower AUC estimates in the high RiskScore group and were negatively correlated with RiskScore (**Figures 11A, B**).

## Discussion

Over the past few decades, the tumor, node, and metastasis (TNM) staging system has played a critical role in the clinical evaluation and treatment of cancer. It provides a framework for describing the clinical course of cancer and categorizing patients into different stages based on factors such as tumor size, lymph node involvement, and distant metastases. Recently, new staging systems have emerged, such as the eighth edition staging system by the American Joint Committee on Cancer. The choice of a staging system is important as it guides treatment selection and prognostic evaluation based on the individual circumstances of the patients. With advancements in molecular biology and immunology, the treatment for HCC has become more diverse, including the use of anti-angiogenic drugs like sorafenib and combination targeted therapy with immune checkpoint inhibitors (24). This diversity highlights the need for better-personalized assessment methods to guide clinical decisions for patients. However, the identification of reliable biomarkers that can accurately identify “personalized” patients with HCC still requires further exploration. We deem it unfeasible to extrapolate this genetic feature to other tumor types due to the variability of biomarkers in different types and subtypes of tumors. Unique biological and genetic features in each tumor type may affect the expression of tumor biomarkers. Consequently, a more comprehensive analysis and assessment are necessary before exploring the suitability of biomarkers in specific tumors. Future research aims to identify more generalized and broadly applicable neutrophil characteristics, which will offer precise and convenient guidance for studying tumor subtypes.

Currently, gene signature models have gained widespread utilization in predicting and diagnosing various diseases, including cancer, cardiovascular disease, and diabetes. These models offer the advantage of simultaneously assessing the expression levels of multiple genes using high-throughput technology, allowing for comprehensive information gathering and a deeper understanding of the underlying biological mechanisms of diseases. By considering multiple genes, gene signature models can mitigate the impact of changes in the expression level of a single gene on the prediction outcomes, thereby improving the accuracy and reliability of the predictions. Recent studies have emphasized the significance of neutrophils as both a prognostic indicator and a target for immune therapy in HCC. However, there is a paucity of studies that accurately predict patient prognosis and determine the efficacy of drug treatment using large-scale machine-learning models specific to HCC. To address this gap, our study aimed to investigate the association between the expression characteristics of neutrophil markers and their potential for benefiting from specific drug therapies in HCC.

Recent advancements in high-dimensional single-cell analyses have provided insights into the heterogeneity of neutrophils present in both the circulation and tumor microenvironments. These studies have revealed variations in transcriptomics and surface protein expression among neutrophils, which can impact the efficacy of immune therapies in patients with cancer (11). The pivotal role of neutrophils in unraveling the heterogeneity of tumors through the identification of molecular markers on their surface has been elucidated. Based on these findings and the potential of neutrophils as effective biomarkers for distinguishing the heterogeneity of tumors, our study aimed to classify patients with HCC based on the expression of neutrophil marker genes at the transcriptome level. The results of the analysis showed significant differences among patients belonging to different subtypes after stratification. Notably, these subtype differences correlated with variations in patient survival, which were further validated across multiple datasets. These findings highlight the feasibility of subtype differentiation based on neutrophil characteristics.

Furthermore, this study further explored the biological differences among the different subtypes of HCC and identified significant differences in signaling pathways by comparing the activity levels of key signal pathways. These findings imply that neutrophils may have a crucial role in the dysregulation of signaling pathways within tumors. However, intriguingly, when examining clinical pathological characteristics, we observed significant differences only in tissue grade among patients classified into different subtypes. On one hand, this observation suggests a potential correlation between subtype classification and the grading of tumors, indicating that neutrophils may serve as a key factor influencing the grading of patients with HCC—a relationship that has not been previously reported. It is important to note that these results may also be influenced by sample size or other factors, warranting further investigation to elucidate the specific underlying mechanisms. Nonetheless, the analysis outcomes of this study vividly demonstrate the presence of distinct “neutrophil characteristics” among patients with different subtypes of HCC.

Based on the feasibility of using “neutrophil characteristics” for the classification of HCC, this study employed univariate Cox regression analysis and a machine learning-based integration program to screen 20 prognosis-related genes derived from characteristic neutrophil genes. Subsequently, a prognostic model was constructed using 10 essential genes. By predicting the expression values of these 10 genes in the TCGA dataset and validation gene set, patients from different datasets were successfully classified into high-risk and low-risk groups. The validation across multiple datasets consistently demonstrated that the high-risk group exhibited a poorer prognosis, while the low-risk group showed a better prognosis.

Furthermore, significant variations were observed in immune cell infiltration levels and immune therapy responses among different cells. Similar research methodologies have been adopted in previous studies to investigate the long noncoding RNA (lncRNA) characteristics of patients with colorectal cancer (CRC), enabling effective evaluation of recurrence, prognosis, chemotherapy response, and immune therapy. These findings are consistent with the results obtained in our study (20). However, lncRNA has inherent challenges such as a low expression level, long and highly variable sequences, and complex detection and measurement processes. In contrast, mRNA-based gene models offer greater clinical translatability and the potential for in-depth research in the future. Additionally, while this study employed multiple datasets for verification, it primarily focused on liver cancer research. In future studies, it is important to validate the generalizability of the model across a broader range of cancer types using additional datasets. Moreover, it is worth noting that this study solely relied on RNA expression data and did not consider other genetic and environmental factors that contribute to the development of liver cancer. Therefore, further refinement of the model is necessary to improve its accuracy by incorporating additional relevant factors. Nevertheless, the existing research results presented in this study confirm and emphasize the feasibility and promising clinical application prospects of the methodology used.

In addition to identifying the “neutrophil characteristics” of HCC, this study also conducted a comprehensive investigation of the gene *RTN3*, which has a significant impact on prognosis. RTN3 is a membrane protein that plays a crucial role in the formation of the endoplasmic reticulum and the regulation of membrane protein acyltransferase activity in normal cells. Extensive research has focused on the role of RTN3 in Alzheimer’s disease, where transgenic mice overexpressing RTN3 show neuroinflammatory abnormalities. Additionally, studies have highlighted the interaction between RTN3 and the oncogene *Ras* within the endoplasmic reticulum. Despite some studies reporting on RTN3 in research on HCC, there remain controversies surrounding its role. For example, certain studies have reported significant upregulation of the levels of RTN3 mRNA and proteins in tumor tissues as a risk factor in risk models (25). Conversely, another study showed that low expression of RTN3 in patients with HCC was significantly associated with poor prognosis, suggesting a potential tumor suppressor role for RTN3 (26). Based on previous studies, it is hypothesized that the role of *RTN3* in HCC is likely influenced by the viral infection status of patients with HCC. On the one hand, studies have reported that the hepatitis B virus (HBV) can induce non-mutational inactivation of the *p53* signaling pathway by interacting with RTN3, which is a crucial mechanism promoting the occurrence and development of HCC. Additionally, a study has demonstrated that RTN3 can directly interact with the non-structural protein of the hepatitis C virus (HCV), leading to the limitation of HCV replication. Therefore, viral infection status may serve as a key determinant of the role of *RTN3* in HCC, although the exact underlying mechanisms still require further investigation. In summary, the research on the role of *RTN3* in tumors remains relatively limited, and the associated mechanisms and biological significance necessitate further investigation. The results of this study indicate that the knockdown of *RTN3* effectively inhibits the proliferation, invasion, and metastasis of tumor cells, thereby confirming the importance of the genes identified in the risk model and providing initial insights into the role of *RTN3* in HCC.

The primary objective of this study is to demonstrate the effective stratification of patients with HCC using neutrophil characteristics of the genes. The application of NDS is theoretically more efficient in clinical decision-making as it primarily involves commonly expressed transcriptome genes. This approach offers cost-effective and personalized molecular feature descriptions to aid in formulating effective treatment strategies and assessing disease progression. However, the study has certain limitations that need to be considered. Firstly, differences in sample sources, data preprocessing, and analysis methods may lead to variations in gene signatures, affecting the stability and reproducibility of predictions. Secondly, gene signature models rely on differences in gene expression levels and may overlook other types of genetic variation, post-transcriptional modifications, and other factors that can influence predictions. Therefore, when applying gene signature models, it is important to acknowledge their limitations and complement them with other biological knowledge and experimental results for a comprehensive analysis. Thirdly, although HepG2 cells have been widely used in the research of HCC, it is essential to recognize that this model may not fully replicate all aspects of human conditions. Future studies will explore the pathogenesis and progression of HCC by using an *RTN3* knockout mouse model. Nonetheless, based on extensive bioinformatics analysis and machine learning algorithms, a stable and powerful feature has been developed to effectively describe the “neutrophil characteristics” of patients with HCC. The NDS model shows promise as a tool for optimizing decision-making and monitoring plans for individual patients with HCC. This study provides a new perspective on understanding the role of neutrophils in HCC and establishes a prognostic model based on NDS, which can serve as a valuable tool for evaluating treatment efficacy and prognosis, offering new ideas and strategies for the treatment and prognosis assessment of patients with HCC.

## Data availability statement

The original contributions presented in the study are included in the article/**Supplementary Material**. Further inquiries can be directed to the corresponding authors.

## Author contributions

QG and XC designed the research. QG and XC performed the research and analyzed the data. FL drafted the paper. QG and YC revised the paper. All authors contributed to the article and approved the submitted version.
