# Supplementary figures and images for: Machine learning-based integration develops a neutrophil-derived signature for improving outcomes in hepatocellular carcinoma
Source: Front Immunol. 2023 Jul 28;14:1216585. doi: 10.3389/fimmu.2023.1216585 (PMC10419218; doi:10.3389/fimmu.2023.1216585)

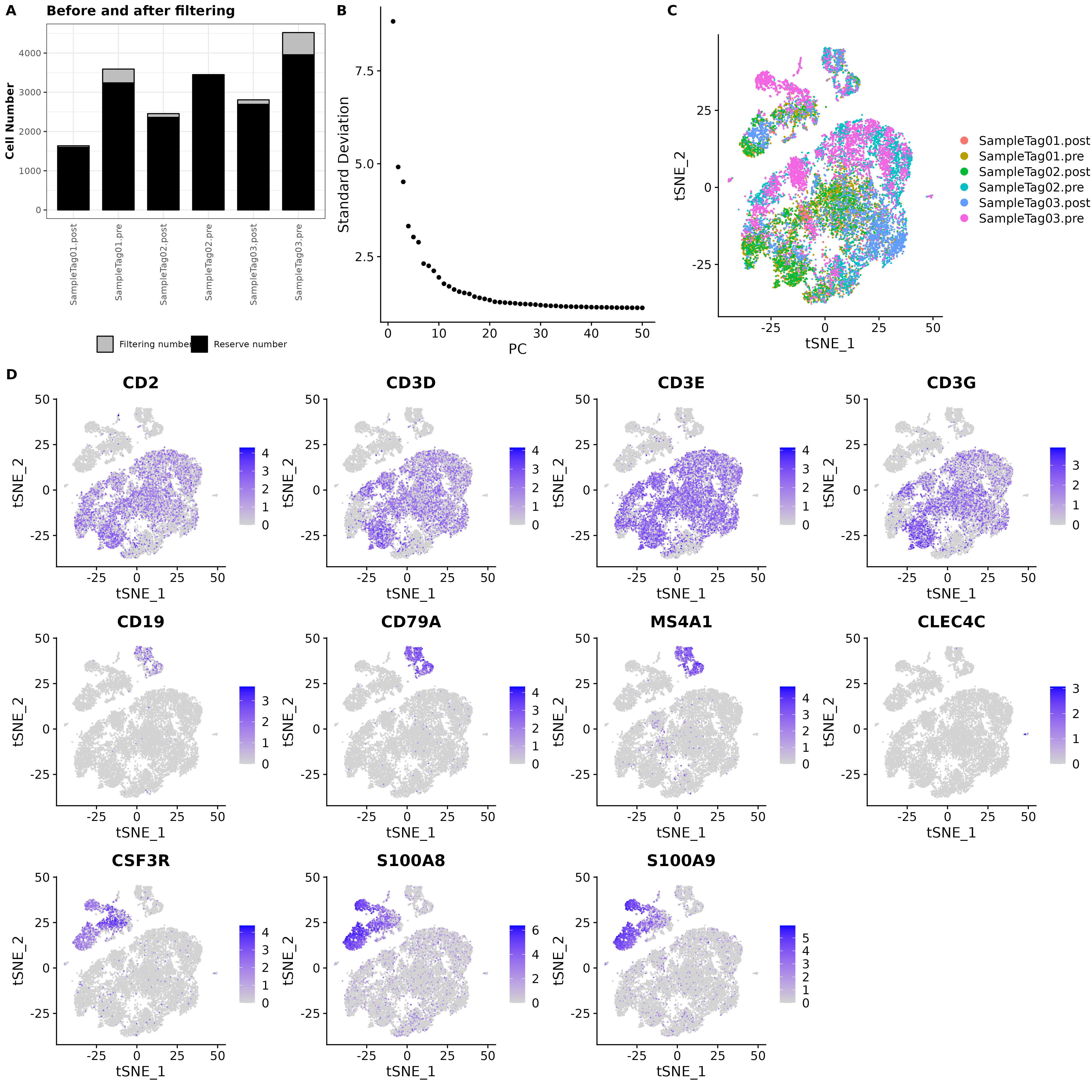

Supplement: Supplementary file 1 [file Image_1.jpeg]

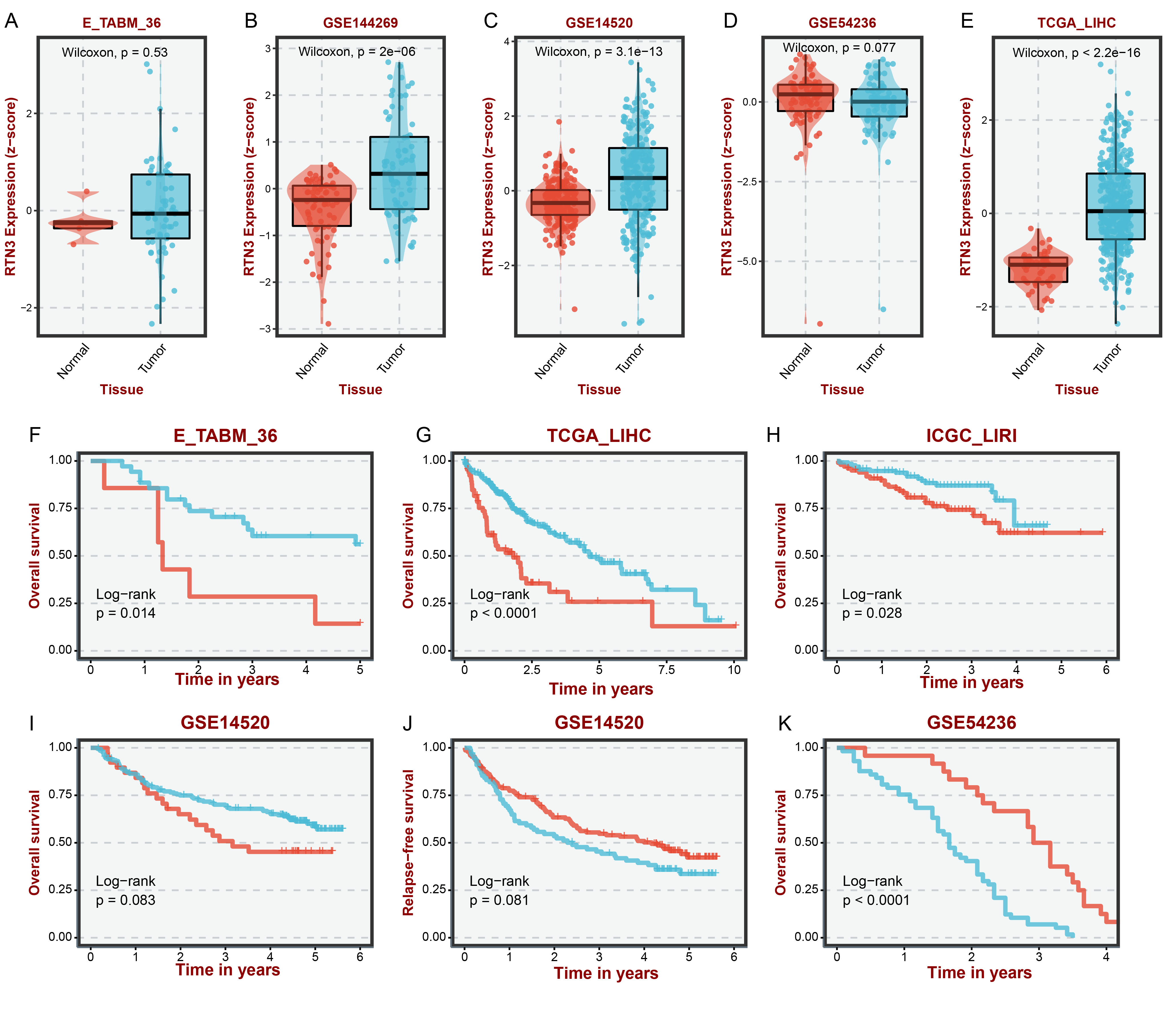

Supplement: Supplementary file 2 [file Image_2.jpeg]
